# Relationship between the difference in electric pulp test values and the diagnostic type of pulpitis

**DOI:** 10.1186/s12903-021-01696-9

**Published:** 2021-07-10

**Authors:** Huachao Sui, Yangyang Lv, Mo Xiao, Liwen Zhou, Feng Qiao, Jinxin Zheng, Cuicui Sun, Jieni Fu, Yufan Chen, Yimeng Liu, Jie Zhou, Ligeng Wu

**Affiliations:** 1 Department of Endodontics, School of Stomatology, Tianjin Medical University, #12 Qi Xiang Tai Road, He Ping District, Tianjin, 300070 China; 2 Department of Endodontics, Wuxi Stomatology Hospital, Jiangsu, China; 3 Department of Oral and Maxillofacial Surgery, School of Stomatology, Tianjin Medical University, Tianjin, China; 4 Department of Endodontics, Tianjin Stomatological Hospital, School of Medicine, Nankai University, Tianjin, China; 5 Department of Stomatology, Wuqing People Hospital, Tianjin, China

**Keywords:** Electric pulp test, Dental caries, Diagnosis, Reversible pulpitis, Symptomatic irreversible pulpitis

## Abstract

**Background:**

According to the diagnosis criteria of the American Association of Endodontists (AAE), sensitive responses to cold and/or heat tests of suspected teeth compared with those of control teeth can be used for the diagnosis of pulpitis, but the role of electric pulp test (EPT) is not mentioned. It is believed that EPT has some limitations in determining the vitality of the pulp. The aim of this study was to explore the association between the difference in EPT values and the differential diagnoses of reversible pulpitis (RP) and symptomatic irreversible pulpitis (SIRP) caused by dental caries.

**Methods:**

A total of 203 cases with pulpitis caused by dental caries were included. A diagnosis of pulpitis was made on the basis of the diagnostic criteria of AAE. Patient demographic and clinical examination data were collected. The EPT values of the suspected teeth and control teeth were measured, and the differences between them were calculated. The correlation between the difference in the EPT values and diagnosis of pulpitis was analyzed using univariate and multivariate logistic regression.

**Results:**

In the 203 cases (78 males and 125 females; 115 cases of RP, 88 cases of SIRP; 9 anterior teeth, 59 premolars, and 135 molars), the mean patient age was 34.04 ± 13.02 (standard deviation) years. The unadjusted (crude) model, model 1 (adjusted for age), model 2 (adjusted for age and sex), and model 3 (adjusted for age, sex, and tooth type) were established for the statistical analyses. In model 3 [odds ratio (OR) = 1.025; 95% confidence interval (CI) 1.002–1.050; *P* = 0.035], the difference in EPT values between RP and SIRP was statistically significant. However, the areas under the curve of predictive probability of the crude model, model 1, model 2, and model 3 were 0.565, 0.570, 0.585, and 0.617, respectively, showing that the model accuracy was low. The *P-*value for the trend in differences between the EPT values as a categorical variable showed that the differences in the EPT values, comparing RP and SIRP, were not statistically significant.

**Conclusions:**

Based on the present data, the difference in EPT values was not sufficient to differentiate RP from SIRP.

## Background

A histopathological examination is the gold standard in assessing pulp status. However, owing to its invasiveness, its use is not recommended to establish clinical diagnosis. Currently, the most common diagnostic methods for detecting pulp state are pulp sensitivity tests (PSTs), including the thermal pulp test and electric pulp test (EPT). The thermal pulp test includes the cold pulp test (CPT) and heat pulp test (HPT) [1–3].

Studies have reported the effectiveness of CPT, HPT, and EPT in evaluating dental pulp vitality [4–6]. Peterson et al. [4] reported that the positive predictive values for CPT, HPT, and EPT were 0.89, 0.48, and 0.88, respectively. Salgar et al. [5] reported that the specificity values of these diagnostic tests were 0.91, 0.84, and 0.90, and that the positive predictive values were 0.89, 0.80, and 0.88 for CPT, HPT, and EPT, respectively. Weisleder et al. [6] reported a sensitivity of 0.76, 0.76, and 0.92; specificity of 0.92, 0.89, and 0.75; positive predictive values of 0.93, 0.90, and 0.83; and negative predictive values of 0.74, 0.73, and 0.87 for the Endo-Ice test, CO_2_ test, and EPT, respectively. These studies indicated that EPT and CPT have similar reliability in judging pulp vitality and are both superior to HPT. As PST results are subjective, combining EPT with a thermal pulp test for a comprehensive diagnosis and mutual verification is warranted.

According to the American Association of Endodontists (AAE), the differential diagnostic criteria for reversible pulpitis (RP) and symptomatic irreversible pulpitis (SIRP) are the presence of spontaneous pain, and lingering pain after cold and/or hot stimuli removal [7]. Although several important signs and symptoms can be used to diagnose SIRP, some are not as straightforward as described in the diagnostic criteria provided by the AAE [8]. For example, some patients have a history of spontaneous pain that lasts for several days, but disappear just before they visit the dentist. Such patients are diagnosed with SIRP depending on their history of spontaneous pain. Additionally, lingering pain after application of thermal stimuli is an important indicator for diagnosing SIRP. However, in clinical practice, when asked about their medical history, many patients do not report an obvious spontaneous or lingering pain, or they do not remember it. Upon clinical examination, only the EPT values of such patients are widely different. Combined with thermal pulp test results, imaging examinations and other oral examinations, the diagnosis of irreversible pulpitis can be established. In the diagnosis criteria of the AAE, the use of EPT for pulpitis is not described. According to our experience, although there is a lack of relevant literature support, EPT results are important in the diagnosis of some atypical SIRP cases.

The thermal pulp test can be used to differentiate RP from SIRP [9, 10], whereas EPT is typically used to evaluate the vitality of the dental pulp [11]. The accuracy of EPT in differentiating the two types of pulpitis has not been reported. Therefore, this study aimed to explore the association between the difference in EPT values for differentiating RP from SIRP for auxiliary differential diagnosis of the two diseases.

## Methods

### Study design and participants

**Fig. 1:**
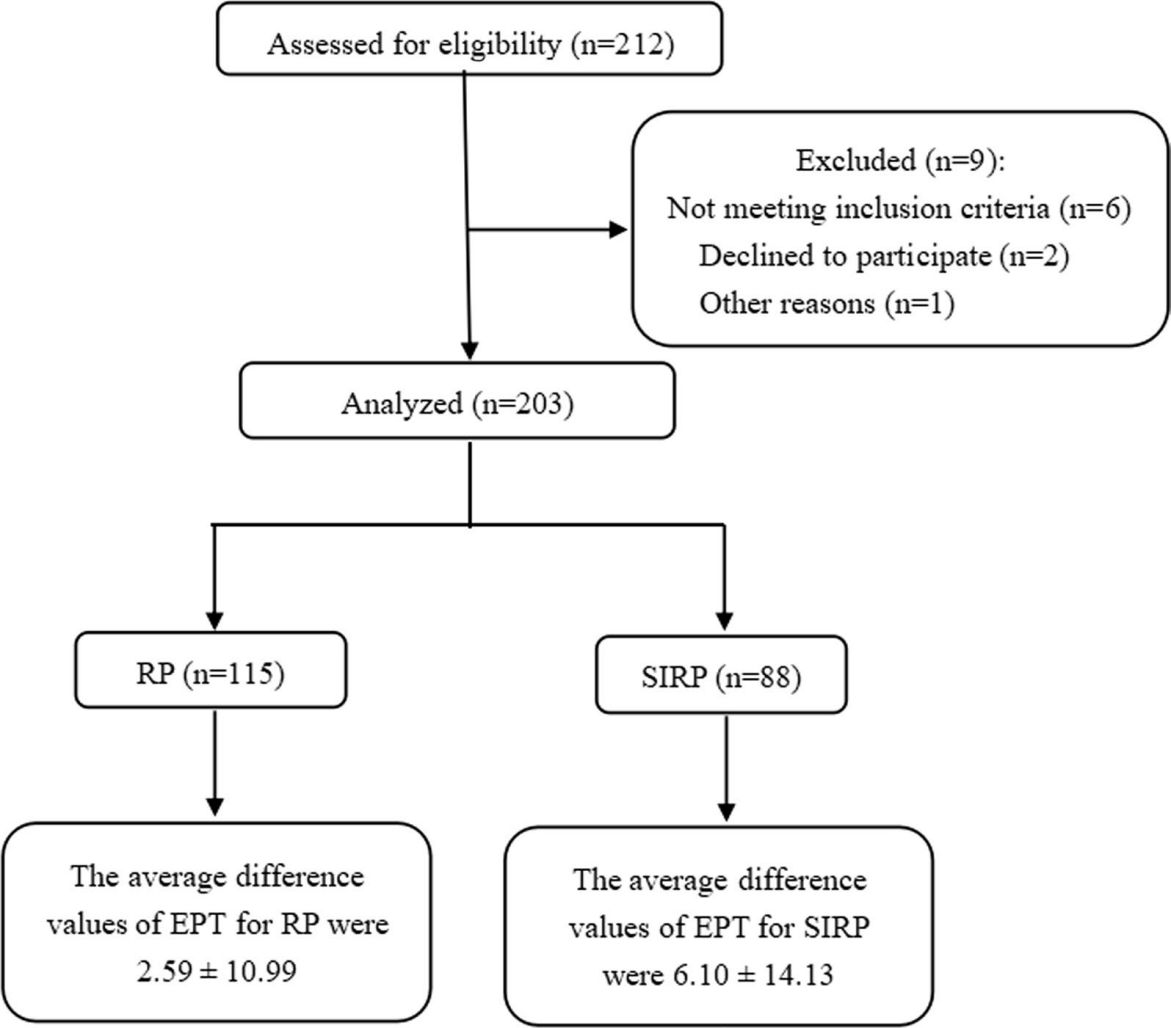
Number of patients with RP and SIRP enrolled and the outcomes in the analysis. *EPT* electric pulp test, *RP* reversible pulpitis, *SIRP* symptomatic irreversible pulpitis

This was a hospital-based retrospective observational study. Patients diagnosed with pulpitis were enrolled at Stomatology Hospital of Tianjin Medical University from September 2017 to December 2019. This study was approved by the ethics committee of the Stomatology Hospital of Tianjin Medical University (project number TMUhMEC2019044). Patient recruitment complied with the following inclusion criteria and exclusion criteria (Fig. 1). Written informed consent was obtained from all subjects. All methods in this study were performed following the institutional review board guidelines.

### Inclusion criteria

1. The diagnostic criteria of pulpitis are met [7]
2. All patients have dental caries
3. Periapical radiographs indicating that the root apices of all teeth had completely developed
4. Teeth without sinuses, swelling, or looseness.

### Exclusion criteria

1. Patients with cardiac pacemakers
2. Young permanent teeth with incomplete apical development
3. Cracked teeth, wedge-shaped defects, traumatized teeth, apical periodontitis, periodontitis, or furcation involvement
4. Patients undergoing orthodontic treatment
5. Antibiotics, analgesics, or anesthetic usage before treatment
6. Systemic diseases such as diabetes, mental disorders, or failure to cooperate with diagnosis.

### Sample size calculation

This was a retrospective study which used the logistic regression method for data analysis. Instead of formulas, empirical method was used to calculate the sample size [12]. This method, also known as the events per variable (EPV) method [13], requires that the number of events (the minimum number of positive and negative events) of the dependent variables not be smaller than the number of independent variables included in the model multiplied by the multiple. In this study, a total of 203 samples were included(88 positive and 118 negative outcomes), with four independent variables to be analyzed. First, the category with fewer dependent variables (n = 88) was selected, the value was divided by 10, resulting in the number of independent variables (n = 8) that could be analyzed in the model. The sample size met the standard, ensuring the robustness of the results.

### Data collection and quality control

**Fig. 2:**
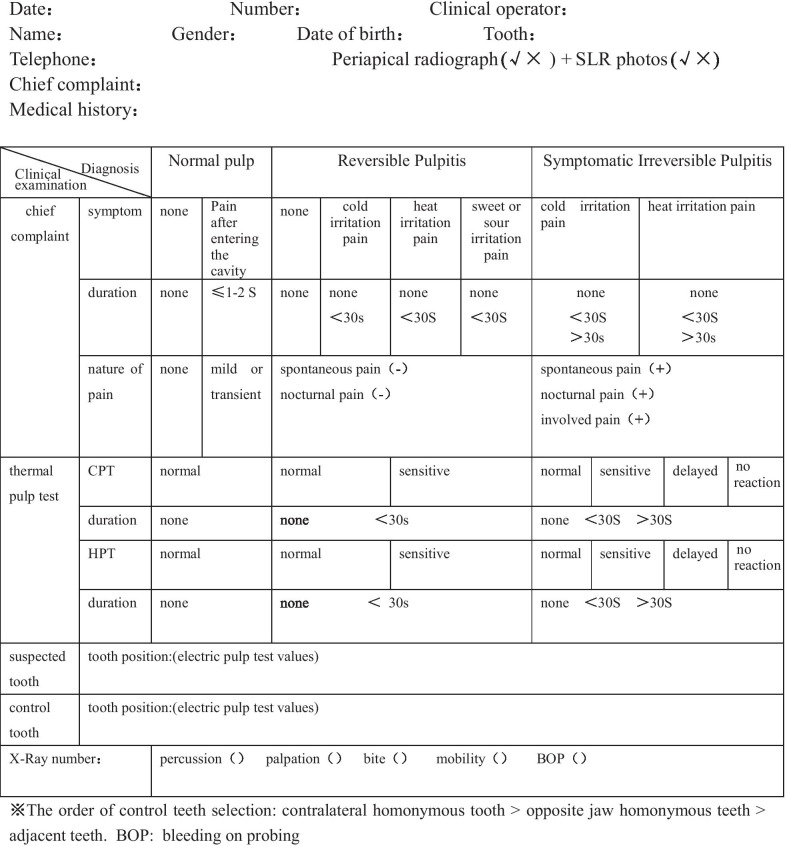
Diagnosis criteria of normal pulp and pulpitis caused by deep dentinal caries according to the American Association of Endodontists (AAE). *BOP* bleeding on probing

The patients were questioned and examined and the results of the examinations were recorded in a table for patients who met the inclusion and exclusion criteria, including the chief complaint, present medical history, CPT, HPT, EPT values, and imaging data (Fig. 2).

Five staff members were involved in the study: a trainer and four inspectors. The trainer was an endodontist with more than 28 years of clinical experience. The inspectors were endodontic graduate students who were trained to conduct clinical interviews and perform standardized oral examinations. We adhered to the principles of quality control during the study, i.e., from the chief complaint to the diagnosis.

### Assessment of candidate predictors

During the PST, control teeth were tested to establish a baseline response, and the patients were informed regarding the experience of a “normal” sensation. This was followed by testing of the suspected teeth. The selection criteria for control teeth included intact teeth, no discomfort, no periodontal pockets, and no previous treatment received. The order of control tooth selection was as follows: contralateral homonymous tooth > opposite jaw homonymous tooth > adjacent tooth. Although the first choice for a control tooth is the contralateral homonymous tooth, sometimes this tooth was lost, had a large restoration, had undergone root canal treatment, etc., which made the tooth unsuitable for obtaining the EPT value. Therefore, as an alternative control tooth, a tooth was selected according to the patient's oral situation.

### Oral examination

The control teeth were tested first, followed by the suspected teeth. Before the three PSTs were conducted, the patient was instructed to raise the left hand when a tingling, warming, or painful sensation was felt during the test. For CPT, the tooth was isolated and dried with cotton rolls. A large #2 cotton pellet with a refrigerant spray (1, 1, 1, 2-tetrafluoroethane; Endo-Frost, Coltene Whaledent, Cuyahoga Falls, OH) at a temperature of 26.2℃ was sprayed and applied to the middle third of the buccal surface of the crown of the tooth for 2 s. Patients then provided their responses.

For HPT, the tooth was isolated and dried with cotton rolls. A thin layer of lubricant was placed on the surface of the tooth and a heated gutta-percha was placed on the middle-third of the buccal surface of the crown of the tooth for 2 s. Patients then provided their responses.

For EPT, the tooth was isolated and dried with cotton rolls. The lip clip was placed on the corner of the lip to complete the circuit. The probe tip of the electric pulp tester (SybronEndo, CA, USA) was coated with toothpaste (Sensodyne, GSK CI), applied to the middle-third of the buccal surface of the crown of the tooth. The test was completed, and the value was recorded when the patient raised a hand. All teeth were tested three times, with an interval of 2 min between the tests. The average values for the suspected and control teeth were calculated, and the difference between these values were obtained.

Percussion, palpation, biting, and radiographic examination were consecutively performed. The diagnosis was finally made according to AAE diagnostic criteria (Fig. 2).

### Outcome definition: criteria for diagnosis of pulpitis

The AAE diagnostic standards for pulpitis [7] are as follows:

- RP: No spontaneous pain, sensitive responses to cold and heat tests compared with those of control teeth, no more than 30 s of lingering pain after the removal of the stimulus, and no significant radiographic changes in the periapical region.
- SIRP: Spontaneous pain, sensitive response to cold or heat tests compared with those of control teeth, more than 30 s of lingering pain after the removal of the stimulus, and no significant radiographic changes in the periapical region.

### Statistical analyses

To perform the statistical analyses for this study, SPSS v.20.0 software (IBM Corp, Somers, NY) and R software were used. Distributions of continuous variables were assessed for normality using the Kolmogorov–Smirnov (n > 2000) and Shapiro–Wilk (n ≤ 2000) tests. Categorical variables were presented as percentages and continuous variables as the median (25%, 75% quantiles). The differences between categories for the clinical diagnosis of pulpitis were assessed using the Kruskal–Wallis test (comparison of > 2 groups) or Wilcoxon rank sum test (comparison of = 2 groups) for continuous and ordinal distributed variables, and the chi-squared test for categorical variables. A linear regression analysis was used to assess the association between the difference in EPT values, and the different types of pulpitis were assessed using a univariate analysis.

For further analysis, multivariate logistic regression was used to analyze the associations between the difference in EPT values and clinical diagnosis of pulpitis after adjustment for potential confounding factors. The receiver operating characteristic curves were generated to assess the predictive probability of the difference in EPT values with regard to the differential diagnosis of the two types of pulpitis. The differences in EPT values were converted into categorical variables, and the *P* values were calculated for the trend. *P* < 0.05 was considered statistically significant.

Restricted cubic splines were also used with four knots at the 5th, 35th, 65th, and 95th centiles to flexibly model the association between the difference in the EPT values and the risk of SIRP.

## Results

In this study, 203 cases (78 males and 125 females; 9 anterior teeth, 59 premolars, and 135 molars) were included, comprising 115 cases of RP and 88 cases of SIRP. The mean age of the patients was 34.04 ± 13.02 (standard deviation [SD]) years.

A linear regression analysis was used to analyze the correlation between age and the difference in EPT values; no significant correlation was observed (*P* > 0.05). An analysis of variance was then used to analyze the effects of sex and tooth type on the difference in EPT values and no significant effect was noted (*P* > 0.05) (Table 1).

**Table 1:** Effect of age, sex, and tooth type on the difference in EPT values on univariate analysis

Variables	βor F	*P* value
Age	0.112	0.113
Sex	0.257	0.612
Tooth type	0.661	0.517

EPT, electric pulp test; β, regression coefficient; F = mean square of intergroup/mean square of intragroup

All continuous variables had a non-normal distribution in the normality test (*P* < 0.05). No significant differences were observed (*P* > 0.05) between age, sex, and clinical diagnosis of pulpitis. A significant difference was observed between tooth type and clinical diagnosis of pulpitis (*P* < 0.05). The average of the difference in the EPT values for RP and for SIRP was 2.59 ± 10.99 (SD) and 6.10 ± 14.13 (SD), respectively, which was statistically significant (*P* < 0.05) (Table 2).

**Table 2:** Demographic and clinical characteristics of the patients with RP and SIRP

Characteristics	RP (n = 115)	SIRP (n = 88)	*P* value
Age, mean (SD), years	34.5 (13.2)	33.5 (12.9)	0.509
Sex, n (%)			
Male	40 (51.3)	38 (48.7)	0.223
Female	75 (60)	50 (40)	
Tooth type			
Anterior teeth	9 (100)	0 (0)	0.023*
Premolars	34 (57.6)	25 (42.4)	
Molars	72 (53.3)	63 (46.7)	
Difference in the EPT values	115 (56.7)	88 (43.3)	0.048*

*RP* reversible pulpitis, *SIRP* symptomatic irreversible pulpitis

^*^P < 0.05

**Fig. 3:**
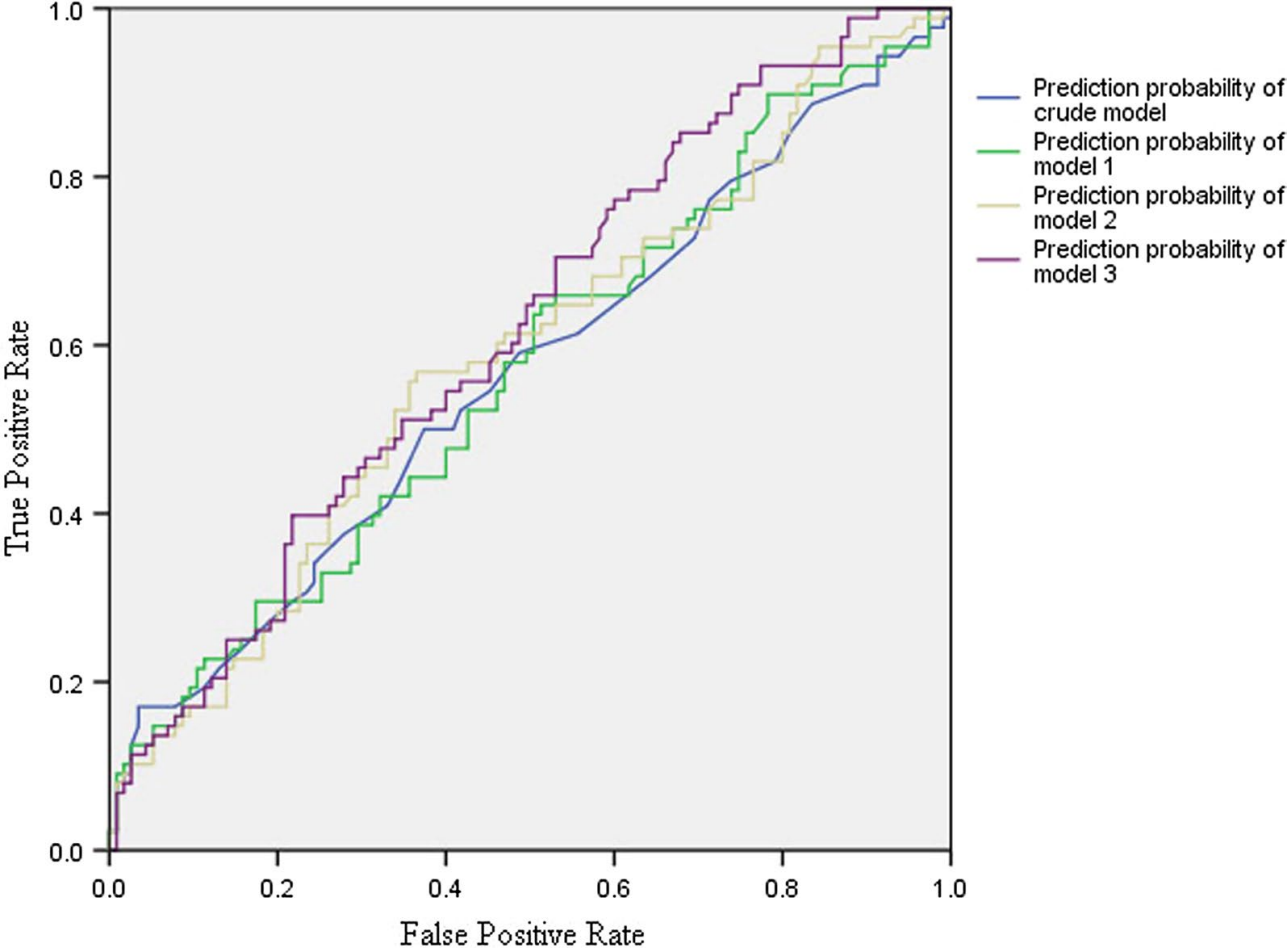
ROC curves for predictive probability of the difference in EPT values for clinical diagnosis of pulpitis in four different models. *ROC* receiver operating characteristic. The area under the curve of predictive probability for model 3 is 0.617

In this study, four models were constructed to analyze the independent effects of the difference in EPT values on the clinical diagnosis of pulpitis in a univariate and multivariate logistic regression. The ORs and 95% CIs are listed in Table 3. In the unadjusted (crude) model, the difference between the difference in EPT values for RP and those for SIRP was not statistically significant. However, in model 1 (adjusted for age), model 2 (adjusted for age and sex), and model 3 (adjusted for age, sex and tooth type), the difference between the difference in EPT values for RP and those for SIRP was statistically significant. The areas under the curve for the predictive probability of the crude model, model 1, model 2, and model 3 were 0.565, 0.570, 0.585 and 0.617, respectively, which showed that the accuracy of these models was low (Fig. 3).

**Table 3:** Relationship between the difference in EPT values and clinical diagnosis of pulpitis in the four models

Variables	Crude model		Model 1		Model 2		Model 3	
	OR (95% CI)	*P*	OR (95% CI)	*P*	OR (95% CI)	*P*	OR (95% CI)	*P*
Difference in EPT values	1.023 (1.000, 1.047)	0.050	1.024 (1.001, 1.048)	0.045*	1.023 (1.000, 1.047)	0.050	1.025 (1.002, 1.050)	0.035*
Difference in EPT values (quartile)								
Q1	Reference	0.498	Reference	0.488	Reference	0.529	Reference	0.448
Q2	1.100 (0.498, 2.431)	0.814	1.114 (0.503, 2.467)	0.798	1.138 (0.513, 2.528)	0.750	1.146 (0.512, 2.565)	0.741
Q3	1.320 (0.610, 2.858)	0.481	1.373 (0.627, 3.008)	0.428	1.299 (0.588, 2.867)	0.518	1.382 (0.618, 3.090)	0.430
Q4	1.800 (0.807, 4.014)	0.151	1.812 (0.812, 4.045)	0.147	1.795 (0.803, 4.017)	0.154	1.904 (0.842, 4.306)	0.122
*P* for the trend	0.133		0.125		0.146		0.109	

Model 1 Adjusted for age

Model 2 Adjusted for age and sex

Model 3 Adjusted for age, sex, and tooth type

Q: quartile of the difference in EPT values

*EPT* electric pulp test, *OR* odds ratio, *CI* confidence interval

^*^P < 0.05

**Fig. 4:**
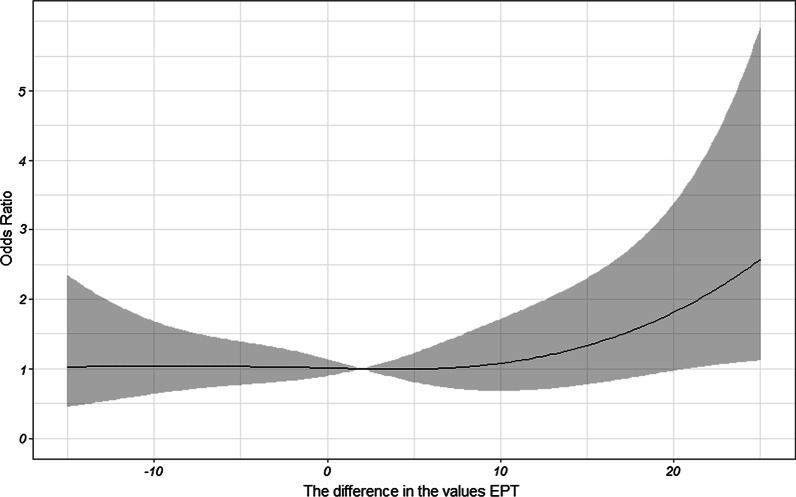
Risk-adjusted, restricted cubic splines with 4 knots of odds ratio for SIRP and the difference in the EPT values. The X axis shows the difference in EPT values; the Y axis shows the odds ratio for SIRP. The black line represents odds ratio for SIRP; the grey area represents the 95% confidence interval. *SIRP* symptomatic irreversible pulpitis, *EPT* electric pulp test

Risk-adjusted, restricted cubic splines with four knots were used to model the probability of SIRP between the difference in the EPT values and the clinical diagnosis of pulpitis, making no underlying assumptions regarding a functional form of the non-linear association between the difference in the EPT values and the clinical diagnosis of pulpitis (*P* = 0.3551, *P* > 0.05) (Fig. 4).

For the purpose of the sensitivity analysis, the difference in the EPT values was converted from a continuous variable to a categorical variable (quartile of the difference in EPT values). The *P* values for the trend of difference in the EPT values as a categorical variable in the four models showed that the difference was not statistically significant (Table 3).

## Discussion

An accurate diagnosis of pulpitis is a prerequisite to form a definite treatment plan. The accuracy of the EPT assessment of pulp vitality has been questioned. Our study assessed the association between the difference in the EPT values between suspected and control teeth and different types of pulpitis diagnosed. However, based on the present data, the difference in EPT values could not be used to differentiate RP from SIRP.

The index used in this study was the difference in EPT values between suspected and control teeth, rather than the numeric readings of EPT for the suspected teeth as reported in other studies [14–16]. The EPT reading is affected by the patient’s age, tooth type, and gender [17–20]. Therefore, the difference in EPT values may be more reliable than the EPT values themselves because the difference in the EPT values uses a normal control tooth as the baseline reference.

Thermal pulp testing is the primary pulp testing method used by many clinicians today. According to the AAE criteria, sensitive responses to cold and/or heat tests compared with those of control teeth can be used for the diagnosis of pulpitis. In addition, an RP or SIRP diagnosis can be established based on the duration of cold and/or heat pain: < 30 s or > 30 s [7]. A tooth sensitive to heat may also manifest spontaneous pain [21], and heat-evoked pain may indicate a deeper inflammation of the pulp of the affected tooth. Typically, a tooth that responds to heat and is relieved with cold stimuli is found to be necrotic. Assessment of pulp neural responses can also be accomplished using EPT [22]. However, a recent systematic review and meta-analysis of the diagnostic accuracy of dental pulp tests revealed that cold, heat, and electric current stimuli are not very accurate in determining pulp vitality [23]. Previous studies also showed that the histological status of the pulp was not associated with the numerical readings of EPT [16, 24].

The low accuracy of PSTs is mainly because of the following reasons: (1) detection principle: CPT, HPT, and EPT are PSTs. The target of these tests is the condition of the pulp sensory nerve; therefore, the result indicates whether the function of the pulp sensory nerve is preserved. The integrity of the pulp blood supply is an important factor in determining pulp vitality. Although it is generally believed that once the pulp blood supply is interrupted, the sensory nerve loses its function [25], the sensory nerve response to stimulation does not mean that the pulp has vitality; (2) PST values are relatively dependent on the patients’ subjective feelings and their mental states. Younger or anxious patients can have false-positive results due to psychological factors [24, 26]. In addition, false-negative results are possible in teeth with incomplete apical development, trauma, root canal calcification, periodontal disease, or in patients undergoing orthodontic treatment [27–30]. Because thermal pulp tests and EPT are not 100% accurate, an accurate diagnosis should not be based on a single test result. Using the three tests combined, one test could verify the previous ones, or the three tests can be mutually verified.

The statistical results of the study were all negative. Univariate logistic regression analyses showed that the average of the difference in EPT values in SIRP (6.10 ± 14.13) was higher than that in RP (2.59 ± 10.99) (Table 2), which is consistent with clinical experience. Clinically, a larger difference in EPT values shows a higher tendency for irreversible pulpitis. Moreover, after the difference in EPT values was converted to a categorical variable from a continuous variable (Table 3), the results also showed that the difference was not associated with the clinical diagnosis of pulpitis. That is, the difference in EPT values could not be used to differentiate RP from SIRP. Therefore, using the difference in EPT values between control and suspected teeth as an independent diagnostic indicator was not feasible. However, this remains a controversial topic. Previous research has shown that EPT and cold testing may accurately diagnose pulp vitality in over 80% of cases [22]. Another study compared the clinical accuracy, reliability, and repeatability of laser Doppler flowmetry (LDF), EPT, and various thermal pulp sensitivity tests and concluded that EPT, CO_2_ test, and LDF were not only reliable, but also the most accurate tests. EPT, though less time-consuming, was found to be less repeatable [31]. The most important point regarding the use of EPT is the interpretation of the results in conjunction with the patient’s history, findings from the clinical examination and radiographs, and the comparison between suspected and control teeth [22, 32]. These two studies only suggested a consideration of the responses in the control teeth; however, evidence was not provided.

In model 3 adjusted for age, sex, and tooth type, the difference in EPT values may help diagnose SIRP (Table 3); however, the area under the curve of the predictive probability of model 3 was 0.617 (Fig. 3). This indicates that the diagnostic accuracy of the difference in EPT values between the suspected and control teeth in RP and SIRP was relatively low.

The result of restricted cubic splines showed that the association between the difference in EPT values and odds ratio (OR) in SIRP was basically linear; however, to some degree, the OR of SIRP increased with the increase in the difference in EPT values (Fig. 4). These two findings were not supported by the relevant literature. However, the results should be further confirmed using multi-center and large sample randomized controlled studies.

Strict quality control was implemented to ensure the reliability of this study. First, several factors influencing EPT were excluded, including cracked teeth, wedge-shaped defects, periodontitis, traumatized teeth, immature permanent teeth, and teeth undergoing orthodontic treatment. Deep periodontal pockets may cause retrograde pulp infection and affect pulp vitality [33], whereas dental trauma may affect the nerve and blood supply of the pulp and further influence the pulp response to EPT [34, 35]. The nerve plexuses of the pulps of immature permanent teeth are incompletely developed, which may render the EPT results unreliable [28, 36–38]. Orthodontic forces would increase the threshold value of teeth in EPT, resulting in a weakened response or no response to EPT [39–41].

Second, three PSTs (CPT, HPT, and EPT) were standardized. The interval between each PST lasted 2 min to avoid the influence of the previous test on subsequent tests. In vitro studies have indicated that the dentin around the pulpal chamber returns to normal temperature 2 min after temperature manipulation [14, 42]. Third, the electric pulp tester used in the study was a unipolar instrument, which only requires a single electrode to contact the tooth surface and to form a complete current circuit through the lip clip [43, 44]; this is convenient for clinical operations and helps to avoid potential clinician errors. Fourth, in all teeth tested in this study, the same toothpaste (Sensodyne, GSK CI) was used as the conducting medium to avoid the influence of different conductive media, which could affect EPT values [15, 20]. Fifth, the probe tip of the electric pulp tester was set on the middle-third of the buccal/labial surface of the crown. Lin et al. [45] reported that the sensitivity differed when the probe tip of an electric pulp tester was placed at different positions; when placed at a position with thinner enamel, sensitivity is improved. Therefore, the placement was fixed at the same position to minimize any errors.

The negative results obtained may be due to several reasons. First, only three potential confounding factors were considered (age, sex, and tooth type); however, other potential confounding factors may also exist. Second, the sample size was very small, as only 203 subjects were enrolled in this study. Moreover, the distribution of tooth types was uneven. Only nine anterior teeth were subjected to EPT during the 2-year study period, which may be associated with a greater concern about the esthetics of anterior teeth; when small caries occurs, patients visit a dentist immediately and get a timely treatment, resulting in fewer EPTs in anterior teeth cases than in other tooth types. Third, the measurement of EPT may not be very accurate. Therefore, it is necessary to confirm the results by increasing the sample size, eliminating the other confounding factors, and balancing the sample stratification.

There were some limitations to this study. The retrospective design inevitably brought about selection bias. The outcomes of the thermal pulp tests and EPT were based mainly on the patient’s subjective feeling; therefore, the feeling described by patient may not meet the definition of what a dentist would define as sensitive or pain. An additional limitation was the relatively small number of samples in our study, especially the anterior teeth, which accounted for only 9 cases. This may be because the anterior teeth are aesthetically involved and patients can notice the lesions early and get treated before the lesions progress to pulpitis. In the future, multi-center and large sample randomized controlled studies are needed to further assess the association between the difference in EPT values between control and suspected teeth and diagnostic types of pulpitis.

## Conclusions

Although there is no evidence that the difference in EPT values can independently predict the diagnosis type of pulpitis, it nevertheless provides us with a clinical clue to assess the state of pulpitis. To some extent, the difference in EPT values is positively correlated with the OR of SIRP. The differential diagnosis between RP and SIRP still mainly depends on the patient's chief complaint and lingering pain after the removal of cold and hot stimuli. A more direct and reliable diagnostic method and tool to properly evaluate the pulpal status is needed.

## Data Availability

The datasets used and analyzed during the current study are available from the corresponding author on reasonable request.

## Abbreviations

RP: Reversible pulpitis
SIRP: Symptomatic irreversible pulpitis
PSTs: Pulp sensitivity tests
EPT: Electric pulp test
CPT: Cold pulp test
HPT: Heat pulp test
AAE: The American Association of Endodontists
SD: Standard deviation
LDF: Laser Doppler flowmetry
